# TLR3 Is a Negative Regulator of Immune Responses Against *Paracoccidioides brasiliensis*

**DOI:** 10.3389/fcimb.2018.00426

**Published:** 2019-01-10

**Authors:** Grasielle Pereira Jannuzzi, José Roberto Fogaça de Almeida, Gustavo P. Amarante-Mendes, Lavínia Maria Dal'Mas Romera, Gilberto Hideo Kaihami, José Ronnie Vasconcelos, Camila Pontes Ferreira, Sandro Rogério de Almeida, Karen Spadari Ferreira

**Affiliations:** ^1^Departamento de Análises Clínicas, Faculdade de Ciências Farmacêuticas da Universidade de São Paulo, São Paulo, Brazil; ^2^Departamento de Imunologia, do Instituto de Ciências Biomédicas, Universidade de São Paulo, São Paulo, Brazil; ^3^Departamento de Química, Instituto de Química, Universidade de São Paulo, São Paulo, Brazil; ^4^Departamento de Biociências da Universidade Federal de São Paulo, São Paulo, Brazil; ^5^Centro de Terapia Molecular e Celular do Departamento de Microbiologia, Imunologia e Parasitologia da Universidade Federal de São Paulo, São Paulo, Brazil

**Keywords:** *Paracoccidioides brasiliensis*, paracoccidioidomycosis, TLR3, CD8^+^T cells, cytotoxic

## Abstract

Toll-like receptors (TLRs) comprise the best-characterized pattern-recognition receptor (PRR) family able to activate distinct immune responses depending on the receptor/adaptor set assembled. TLRs, such as TLR2, TLR4 and TLR9, and their signaling were shown to be important in *Paracoccidioides brasiliensis* infections. However, the role of the endosomal TLR3 in experimental paracoccidioidomycosys remains obscure. *In vitro* assays, macrophages of the bone marrow of WT or TLR3^−/−^ mice were differentiated for evaluation of their microbicidal activity. *In vivo assays*, WT or TLR3^−/−^ mice were infected intratracheally with *Paracoccidioides brasiliensis* yeasts for investigation of the lung response type induced. The cytotoxic activity of CD8^+^ T cells was assessed by cytotoxicity assay. To confirm the importance of CD8^+^ T cells in the control of infection in the absence of tlr3, a depletion assay of these cells was performed. Here, we show for the first time that TLR3 modulate the infection against *Paracoccidioides brasiliensis* by dampening pro-inflammatory response, NO production, IFN^+^CD8^+^T, and IL-17^+^CD8^+^T cell activation and cytotoxic function, associated with granzyme B and perforin down regulation. As conclusion, we suggest that TLR3 could be used as an escape mechanism of the fungus in an experimental paracoccidioidomycosis.

## Introduction

The receptors of the innate immune system recognize microbes through germline-encoded pattern-recognition receptors (PRRs) and initiate the mechanisms responsible to eliminate potentially infectious threats (Kumar et al., 2011; Brubaker et al., 2015). Most PRRs can be classified into one of five families based on the protein domain homology. Their activation also initiates non-transcriptional responses, such as the induction of phagocytosis, autophagy, cell death, and cytokine production (Drummond and Brown, 2011; Deretic et al., 2013; Lamkanfi and Dixit, 2014).

PRRs detect molecular components of microbial pathogens, which are referred to as pathogen-associated molecular patterns (PAMPs). The toll-like receptor (TLR) family consists of well-characterized PPRs (Kawai and Akira, 2010; Ruiz et al., 2015). TLRs are widely expressed in immune cells and non-immune cells and can recognize a broad array of microorganisms (Abe et al., 2007). During the past few years, several studies have demonstrated a role for TLRs in the recognition of fungal pathogens, such as *Paracoccidioides brasiliensis, Candida albicans, Cryptococcus neoformans* and *Aspergillus fumigatus* (Braedel et al., 2004; Netea et al., 2004; Calich et al., 2008). In this context, both, extracellular and intracellular domains of TLRs are important in the activation of the innate immune response. TLR3 is an endosomal PRR that recognizes pathogen-specific PAMPs that trigger activation of NFκB and IRF3 to induce cytokine production in infected cells (Takeuchi and Akira, 2010). These receptors are regulators in endosomes, and we know that TLR3 recognizes double stranded RNA (dsRNA) and heterologous dsRNA (Alexopoulou et al., 2001; Kawai and Akira, 2008; Liu et al., 2008; Carvalho et al., 2012). In fungal infection, Carvalho and co-workers showed that mice treated with fungus-derived RNA induced an effective immune response against Aspergilloses, via TLR3 (Carvalho et al., 2012). However, the role of TLR3 in paracoccidioidomycosis (PCM), which is a deep mycosis, is not clear.

PCM is a systemic granulomatous infection caused by the four distinct phylogenetic lineages of *Paracoccidioides* ssp (Matute et al., 2006; Teixeira et al., 2009; Bocca et al., 2013). Our previous studies showed that interaction between *Paracoccidioides brasiliensis* and pulmonary dendritic cells induces IL-10 production and TLR2 expression. These results suggested TLR2 and IL-10 might be involved in the susceptibility to *Paracoccidioides brasiliensis* (Ferreira et al., 2007).

Considering that interaction of host cells with whole pathogens is a very complex process, it is believed that the resulting adaptive immune response (Th1/Th2/Th17) (Gil et al., 2016) depends on the integrative engagement of different PRRs and their related PAMPs. Interesting enough, we have shown that TLRs collaborate with other PRRs like dectin-1 and dectin-2 in PCM (Romera et al., 2017). Nevertheless, to gain novel insights on a particular host-pathogen interaction it is crucial to first dissect individual signaling pathways. Here, we investigated the function of TLR3 in *Paracoccidioides brasiliensis* pulmonary infection. Our results showed, for the first time, that the TLR3 is a negative regulator of CD8 cytotoxic T cell-mediated immune response and protection against experimental PCM.

## Materials and Methods

### Animals and Ethics Statement

For this study, male TLR3^−/−^ and wild type C57BL/6 (WT) mice (8–12 week-old) were obtained from specific pathogen-free conditions facility of the University of São Paulo. The Committee on the Ethics of Animal Experiments at the University of São Paulo approved the protocol (CEUA/449). The mice were housed conformed to institutional guidelines for animals and welfare.

### Paracoccidioides Brasiliensis Strain

To the infections experiments, we used the yeast of *Paracoccidioides brasiliensis* strain 18 (Pb18) (a highly virulent strain) were grown on Sabouraud agar (Becton, Dickinson and Company, Le Pont de Claix, France). The viability of the yeast cells was determined using trypan blue. We used cell populations with viabilities higher than 90%.

### Macrophages From Bone Marrow

Macrophages from TLR3^−/−^ mice were differentiated from bone marrow as previously described (Davis et al., 2012, 2013). The cells were derived by culturing bone marrow freshly isolated from the femurs of mice in medium consisting of RPMI supplemented with 20% fetal calf and 50 ng/mL recombinant M-CSF for seven days. Cultures were nourished with additional M-CSF-containing media on the third day of culture.

### Phagocytosis Test and Nitric Oxide Production

For the phagocytosis test, macrophages (2 × 10^5^ cells) were cultured on round, glass coverslips in 24-well plates. After 4 h of incubation with Pb18 yeast (ratio 1:1) at 37°C, the plate was rinsed with PBS non-internalized yeast cells and stained with Giemsa stain. A mean of 100 macrophages was counted to determine the phagocytic index, which was calculated as the percentage of phagocytic cells in relation to the total number of cells multiplied by the mean number of internalized or bound particles. To NO production, the supernatant fluids were analyzed by Sievers Nitric oxide analyzer (NOA 280).The fungicidal activity of the macrophages was described elsewhere (da Silva et al., 2006). To CFU assay, the macrophages were incubated with Pb18 (ratio 1:1) for 4 h at 37°C. After incubation, the cells were lysed by adding 100 mL of sterile water for 30 min. Then, the supernatant fluids and the lysate were pooled and plated on brain-heart infusion agar supplemented with 5% fetal calf serum to determine yeast cell viability. The plates were incubated at 37°C, and the number of colony-forming units were counted after 15 days.

### Murine Infection

TLR3^−/−^ mice (7/group) were challenged with an intratracheal inoculation of 1 × 10^6^ Pb18 yeast cells (Inaba et al., 1992). After 30 days of infection, all animals were sacrificed, and the lungs were removed for colony forming unit (CFU) analysis. As a control, we used C57BL/6 (WT) mice. The CFU was counted as previously described (Singer-Vermes et al., 1992).

### Flow Cytometry Assay

To analyze the phenotype of the cells in the lung after infection (30 days), the total cells were obtained and analyzed by flow cytometry with a FACS Canto II (Becton Dickinson). To determine the expression of TCD4^+^ and TCD8^+^ in the cells, we used labeled mAbs against mouse PE CD3e (145C11), FITC CD4 (L3T4 6K 1.5) and APC CD8 (53–6.7). To analyze the IL-17^+^CD8^+^ and IFN-γ+ CD8^+^ T cells, the lungs cells were incubated with Brefeldin A for 12 h. After that, the CD8^+^T cells were stained with PerCP CD3e (145–2C11) and APC CD8 (53–6.7) and then we used the fixation and permeabilization kit (eBioscience) according to the manufacturer's protocol. The FITC-IFN-γ and PE-IL-17 antibody were added and then the CD8^+^T cells were analyzed by flow cytometer. To analyze Treg cells, the cells were stained with PerCP CD3e (145-2C11), PE CD4 (H129.19), PE-Cy7 CD25 (PC61.5), and then we used the fixation and permeabilization kit (eBioscience) to stain intracellular with Alexa Fluor 488 FoxP3 (150D/E4). All antibodies were obtained from BD Biosciences (San Jose, CA). The flow cytometry data were analyzed using FlowJo. Fluorescence-minus-one (FMO) tubes were used as additional controls.

### Cytotoxicity Assay

To cytotoxic assay, CD8^+^T cells were obtained from TLR3^−/−^ or WT mice infected for 30 days. The CD8^+^T cells were purified from the spleen through positive selection using a mAb specific (mouse-Miltenyi Biotec) and then, the cells were analyzed by flow cytometry with a FACS Canto II (Becton Dickinson) to check the purity. We used labeled mAbs against mouse PerCP CD3e (145C11) and APC CD8 (53-6.7). All antibodies were obtained from BD Biosciences (San Jose, CA). On the other hand, macrophages derived-bone marrow was obtained from WT mice and labeled with CFSE (carboxyfluorescein diacetate succinimidyl diester—Molecular Probes, Eugene, Oregon, USA) at 10 μM. After that, macrophage cells (1 × 10^5^ cells/ml) were cultivated in a 24-well plates overnight. The cells were incubated with Pb18 yeast (ratio 1:1) at 37°C for 4 h. Then, the lymphocytes CD8^+^T cells were added (5 × 10^5^ cells/mL) and incubated for 4 h. The macrophages viability were verified with Propidium Iodide Staining Solution (PI) (BD Bioscience) and analyzed by flow cytometry with a FACS Canto II (Becton Dickinson).

### Viability of Yeast Assay

The viability of yeast was realized with supernatant of cytotoxic assay, thus we incubate overnight with live yeast or heat killed (121°C for 30 min). To the positive control death was used R10 culture medium and yeast heat killed and as negative control death was used R10 culture medium and lives yeast. After incubation time, we add 1 uL of PI and analyzed the viability of yeast by Fluorescence microscopy.

### Measurement of Cytokines

Cytokine levels were measured with an ELISA assay (eBiosciences kits) according to the manufacturer's protocol. After infection, the lungs were collected, weighted and the homogenate from the organ was filtered in cell stainer 70μm and centrifuged at 1,400 × g at 4°C for 10 min, and the supernatants were assayed for IFN-γ, IL-12, IL-6, IL-17, IL-10, and IL-1 β. The cytokines content were normalized by organ weight.

### CD8 T Cells Depletion

Depletion of CD8 + T cells was performed by the treatment of TLR3^−/−^ with the monoclonal antibody 53.6.7 (Anti-CD8). On days−7,−5,−3, and−1 prior to infection with Pb18 yeasts, the mice were injected intraperitoneally with the dosage of 0.5 mg of Anti-CD8 or control IgG rat. After 2 days of infection, each mouse received another 0.5 mg dose of Anti-CD8 or IgG rat control. The treatment was maintained every 3 days until 30 days of infection. The CD8 + T cell depletion protocol was based on the article by Vasconcelos et al. (2012).

### Statistics

Prism 5 (GraphPad Inc.) was used for all statistical analyses. The data were compared using either a two-way analysis of variance (ANOVA) followed by Bonferroni's multiple comparison tests or an ANOVA followed by the Tukey-Kramer test (Zar, 1984). All data are represented as the mean and standard deviation (SD).

## Results

### TLR3 Negatively Regulates Fungicidal Activity of Macrophages Against *P. brasiliensis*

**Figure 1:**
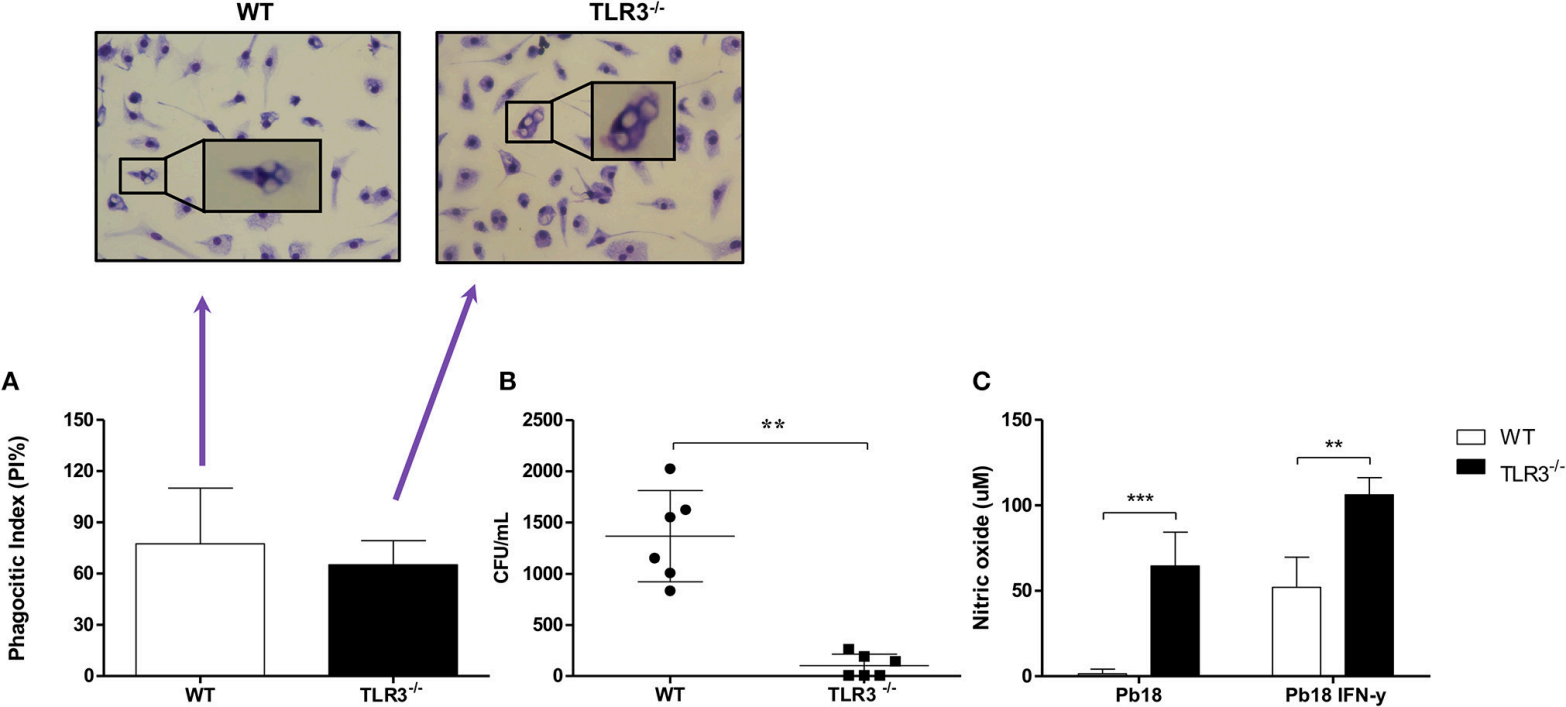
TLR3^−/−^ macrophages induce a higher microbicidal activity *in vitro*. Macrophages from WT and TLR3^−/−^ mice (2 × 10^5^ cells) were cultivated with 2 × 10^5^ of Pb18 yeast. After 4 h, **(A)** the PI was evaluated. In addition, **(B)** the cells were lysed and the CFU were counted. **(C)** The supernatant of **(A)** was collected and NO production was measured from macrophages activated or not with IFN-γ (10 ng/mL). Data are means of seven independent experiments (^**^P < 0.001 and ^***^P < 0.0001).

Macrophages are antigen presenting cells that are capable of recognizing and capturing yeast cells. To analyze the phagocytosis process, macrophages from WT or TLR3^−/−^ mice were cultivated with Pb18 yeast cells for 4 h and the phagocytic index (PI), the fungal burden and NO production were analyzed. Our results show no significant difference in PI between macrophages from WT or TLR3^−/−^ mice (Figure 1A). However, the fungicidal activity of TLR3^−/−^ macrophages is increased compared to WT macrophages, as observed by significant decrease of fungal viability, measured by CFU obtained after macrophage lysis (Figure 1B). Interestingly, the higher fungicidal activity of TLR3^−/−^ macrophages is associated with a significantly higher NO production (Figure 1C). Therefore, TLR3 signaling in response to *P. brasiliensis* reduces anti-fungal activity of macrophages *in vitro*.

### TLR3 Deficiency Improved Control of *Paracoccidioides brasiliensis* Infection

**Figure 2:**
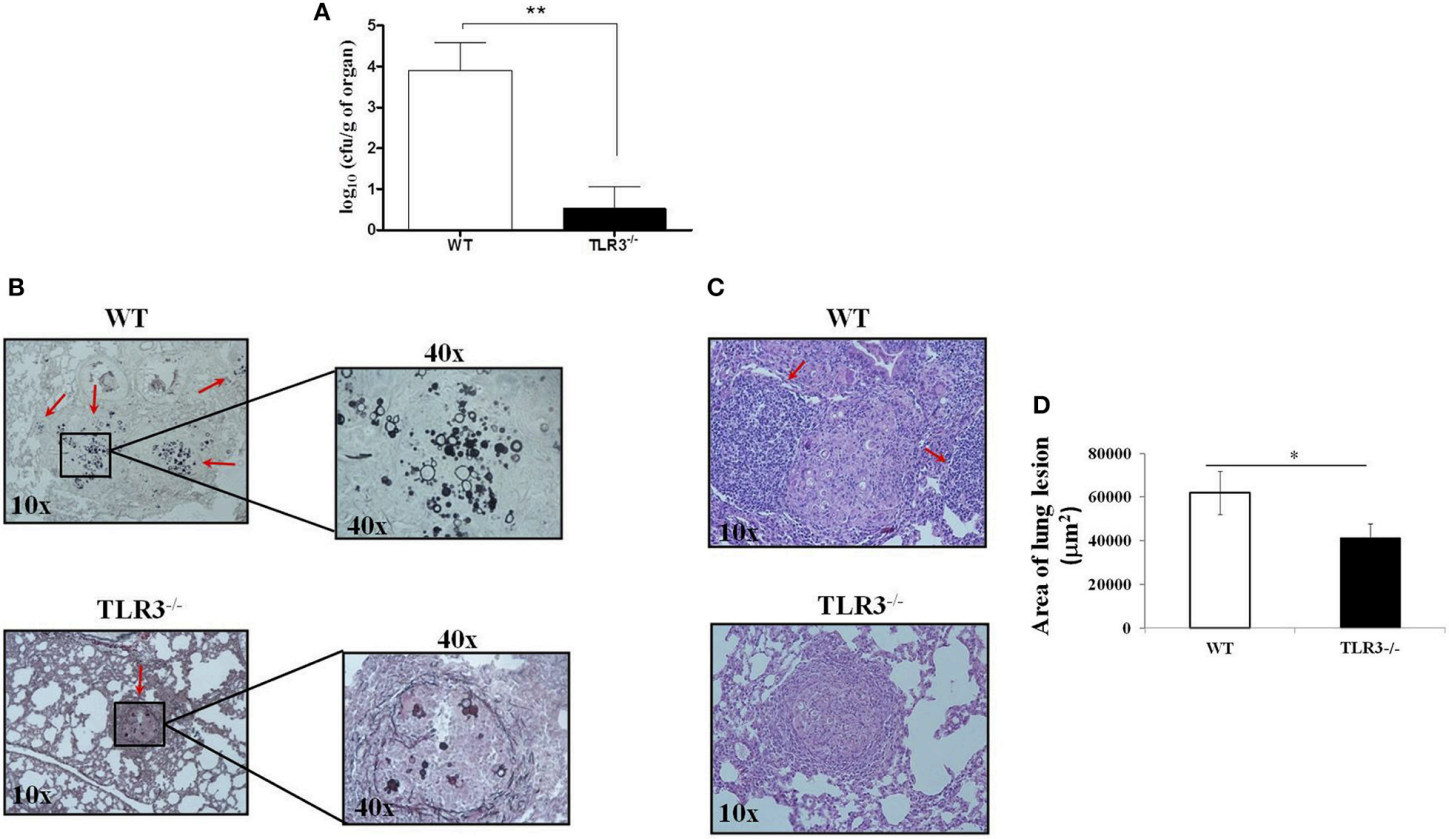
TLR3-3 induce a decreased of fungal burden. WT and TLR3^−/−^ mice (7 animals/group) were infected with 1 × 10^6^ yeast cells of Pb18 strain. After 30 days, the lung were obtained and we evaluated: **(A)** CFU and **(B)** histopathology (*Grocott* stain) from WT and TLR3^−/−^ mice, **(C)** granulomatous process in WT and TLR3^−/−^ mice. In B, red arrow indicates the fungus and in C, indicate the inflammatory cells. **(D)** Total areas of lesions in the lung of mice (*n* = 10). Dates are means of five independents experiments (**P < 0,001 and *P < 0.05).

To analyze the relevance of our findings in experimental PCM, TLR3^−/−^ and WT mice were infected with Pb18 yeast cells using the intratraqueal route. After 30 days, the lungs were harvested, and the CFU and histopathology were evaluated. In line with our previous data, we observed a significant decreased in CFU obtained from TLR3^−/−^ compared to WT mice (Figure 2A). In addition, Grocott stain of lung tissue preparation obtained from TLR3^−/−^ mice revealed lower numbers of *Paracoccidioides brasiliensis*, as compared with the WT mice (Figure 2B). Furthermore, TLR3^−/−^ lungs showed lower cellular infiltrate compared to the WT lungs (Figure 2C, respectively). The lungs of the WT mice presented greater lesion area when compared to the TLR3 mice (Figure 2D). These results suggested that TLR3 controls the fungicidal response to *Paracoccidioides brasiliensis* also *in vivo*.

### TLR3 Deficiency Recruited TCD8^+^ Cells in the Lung

**Figure 3:**
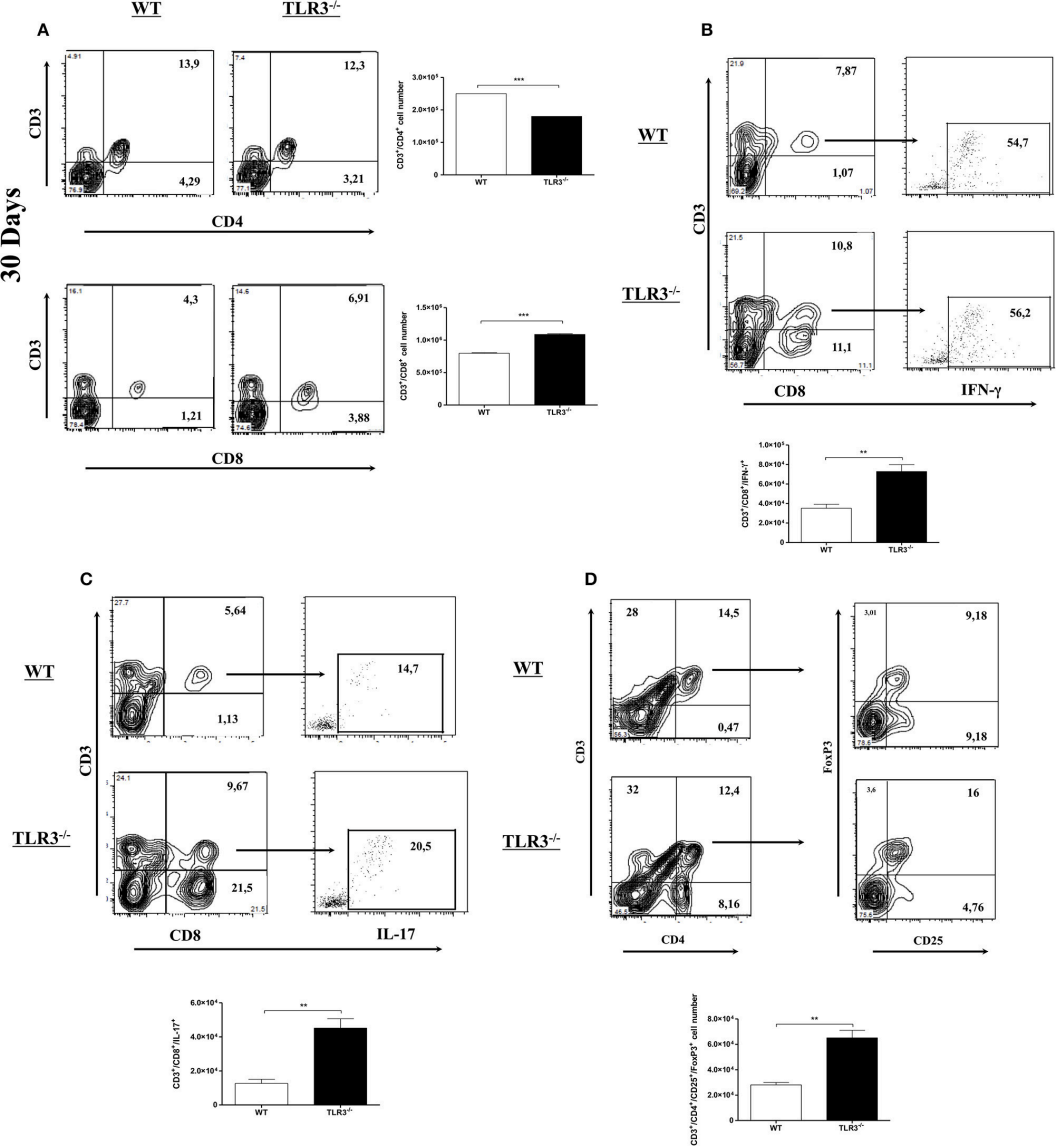
CD8^+^ T cells increased in the lung of TLR3^−/−^ mice. WT and TLR3^−/−^ mice (seven/group) were infected with 1 × 10^6^ of Pb18 yeast for 30 days and **(A)** total CD4^+^T and CD8^+^T cells from lung tissue were investigated by flow cytometry. **(B)** the IFN-g and **(C)** IL-17 intracellular cytokines produced by CD8^+^T cells were investigated by flow cytometer and the results were measured from mix of lung from seven animals. **(D)** Total Treg cells from lung tissue were investigated by flow cytometry. Data are expressed, as the numbers of cells obtained with specific Ab. Results are representative of three independent experiments. (**P < 0,001 and ***P < 0.0001).

Infection with Pb18 yeast can induce the recruitment and activation of different cell types in the lung. Because T cells were shown to be important in the control of experimental PCM, we decided to investigate CD4^+^ and CD8^+^ T cells in the lung of TLR3^−/−^ and WT mice after 30 days of infection. Total cells from the lung were obtained, and the number of CD4^+^ and CD8^+^ T cells was analyzed. Figure 3A shows a decrease in CD4^+^ T cells, but a significant increase of CD8^+^ T cells (Figure 3B) in the lungs of TLR3^−/−^, when compared to WT infected mice. The number of dendritic and NK cells was also evaluated, however no difference between the mouse lines was observed (Supplementary Figures 1A,B). In addition, we observed an increase of IL-17^+^CD8^+^T cells and IFN-γ^+^CD8^+^T cells (Figure 3C). Although the number of total TCD4 cells was lower in the TLR3^−/−^ mice, the production of both IFN and IL-17 by these cells was superior when compared to WT mice (Supplementary Figures 2A,B). To analyze the population of T regulatory cells (Treg), the cells were stained with a combination of anti-CD4, anti-CD25, and anti-FoxP3. Our results demonstrated the presence of a higher amount of Treg (CD25^+^/FoxP3^+^) in lung from TLR3^−/−^ mice, when compared with WT mice (Figure 3D).

### TLR3^−/−^ Infection in Mice Induced Pro-Inflammatory Cytokines

**Figure 4:**
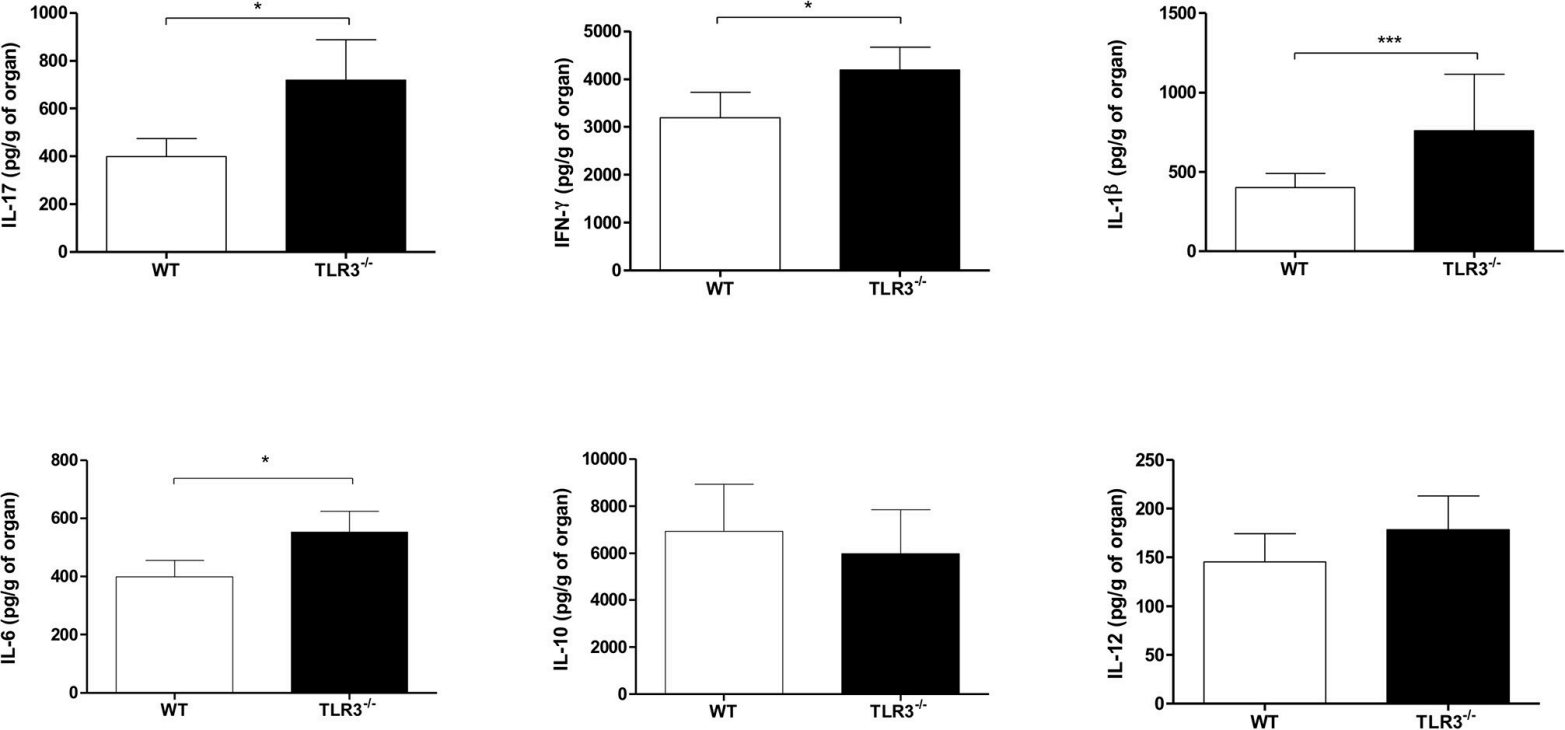
Modulation of cytokines in the lung and lymph nodes from TLR3^−/−^ mice. WT and TLR3^−/−^ mice (seven/group) were infected with 1 × 10^6^ of Pb18 yeast for 30 days. After that, the cytokines IFN-γ, IL-17, IL-12, IL-1β, IL-6 e IL-10 from the lung were measures by ELISA assay. Results are representative of four independent experiments. (*P < 0.05 and ***P < 0.0001).

Since we observed that WT mice presented a higher inflammatory infiltrate in response to *P. brasiliensis* infection compared to TLR3^−/−^ mice, we measured the production of pro-, and anti-inflammatory cytokines in the lung of these two mice strains. Our results showed a significant increase in IL-17, IFN-γ, IL-1β, and IL-6 in the lungs of TLR3^−/−^ mice, but no difference in IL-10 and IL-12 production (Figure 4).

### TLR3 Negatively Regulates Number and Function of CD8^+^T Cells in PCM

**Figure 5:**
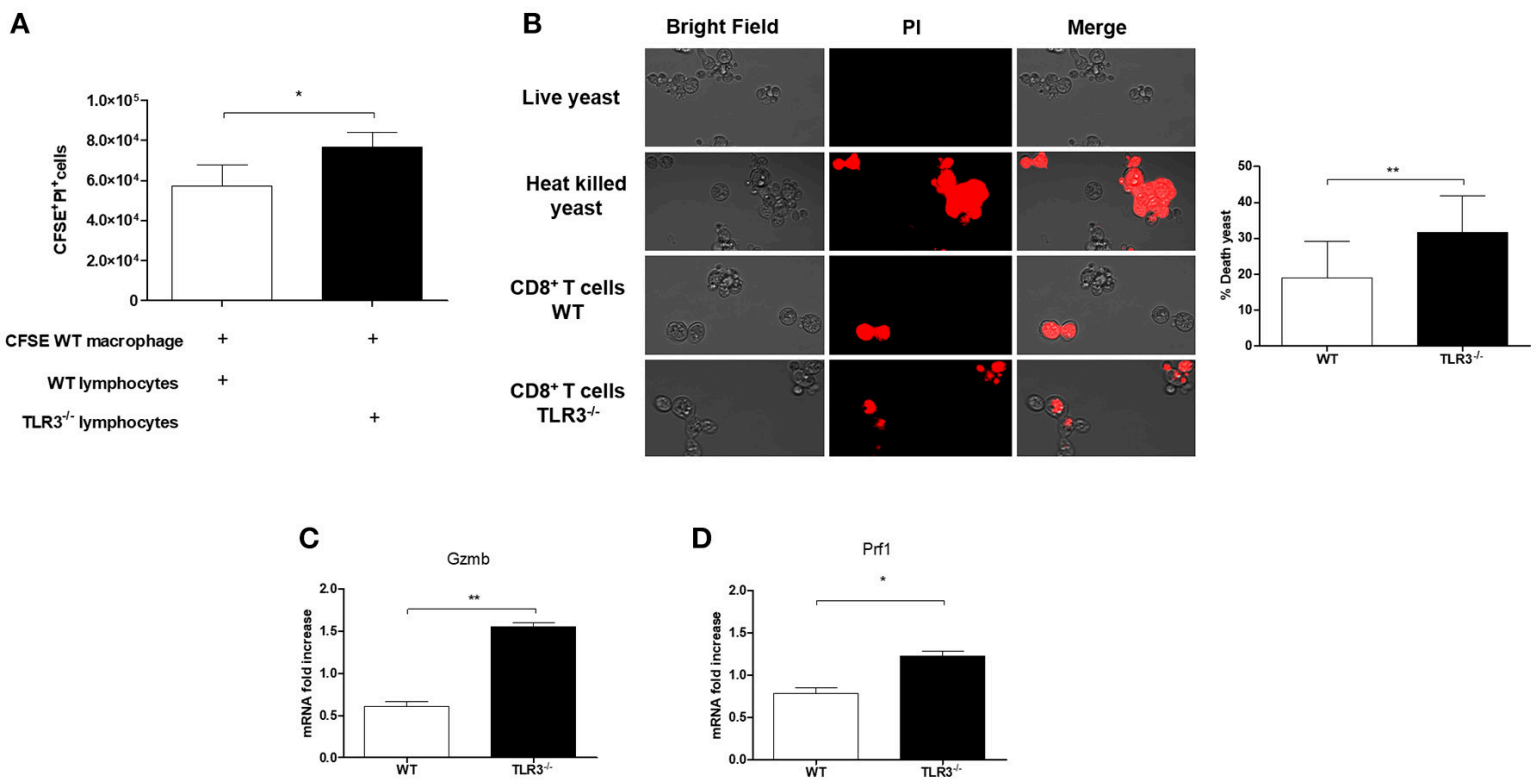
Cytotoxicity of CD8^+^ T cells from TLR3^−/−^ mice. **(A)** Cytotoxicity assay was performed. Macrophages derived by bone marrow of WT mice were stained with CFSE and infected with 1 × 10^5^ Pb18 yeast. After 4 h the lymphocytes WT or TLR3^−/−^ were added in the culture (5 × 10^5^cells) and the cytotoxicity was measured with the dye PI by flow cytometry. Results are representative of three independent experiments. (*P < 0.05). Yeast activity of culture supernatants from CD8^+^ T cells exposed as in **(B)**. Live yeast were cultured overnight with the supernatant and then incubated with dye PI before examination by fluorescence microscopy. **(C)** Relative expression of GZMB and **(D)** Prf1 by RT-PCR in CD8^+^T cells exposed to macrophages in **(A)**. (*P < 0.05 and **P < 0.001).

CD8^+^T cells plays an important role in the resistance to PCM. Therefore, we investigated the cytotoxic function of these cells against *Paracoccidioides brasiliensis* in both WT and TLR3^−/−^ mice. As showed in Figure 5A, we observed a decreased in viability of macrophages infected with Pb18 and cultivated in presence of CD8^+^T cells mainly when we used TLR3^−/−^ lymphocytes. Also, we can see by microscopy and by percentage, an increase of the death yeast cells when we analyze the supernatant of CD8^+^ T cells from TLR3^−/−^, as we showed in Figure 5B. In addition, the results showed an increase of granzyme (*gzmb*) and perforin (*prf1*) by CD8^+^T cells from TLR3^−/−^, as compared with the same cells from WT mice (Figures 5C,D).

### CD8^+^ T Cells Depletion Induce an Increase of Fungal Burden

**Figure 6:**
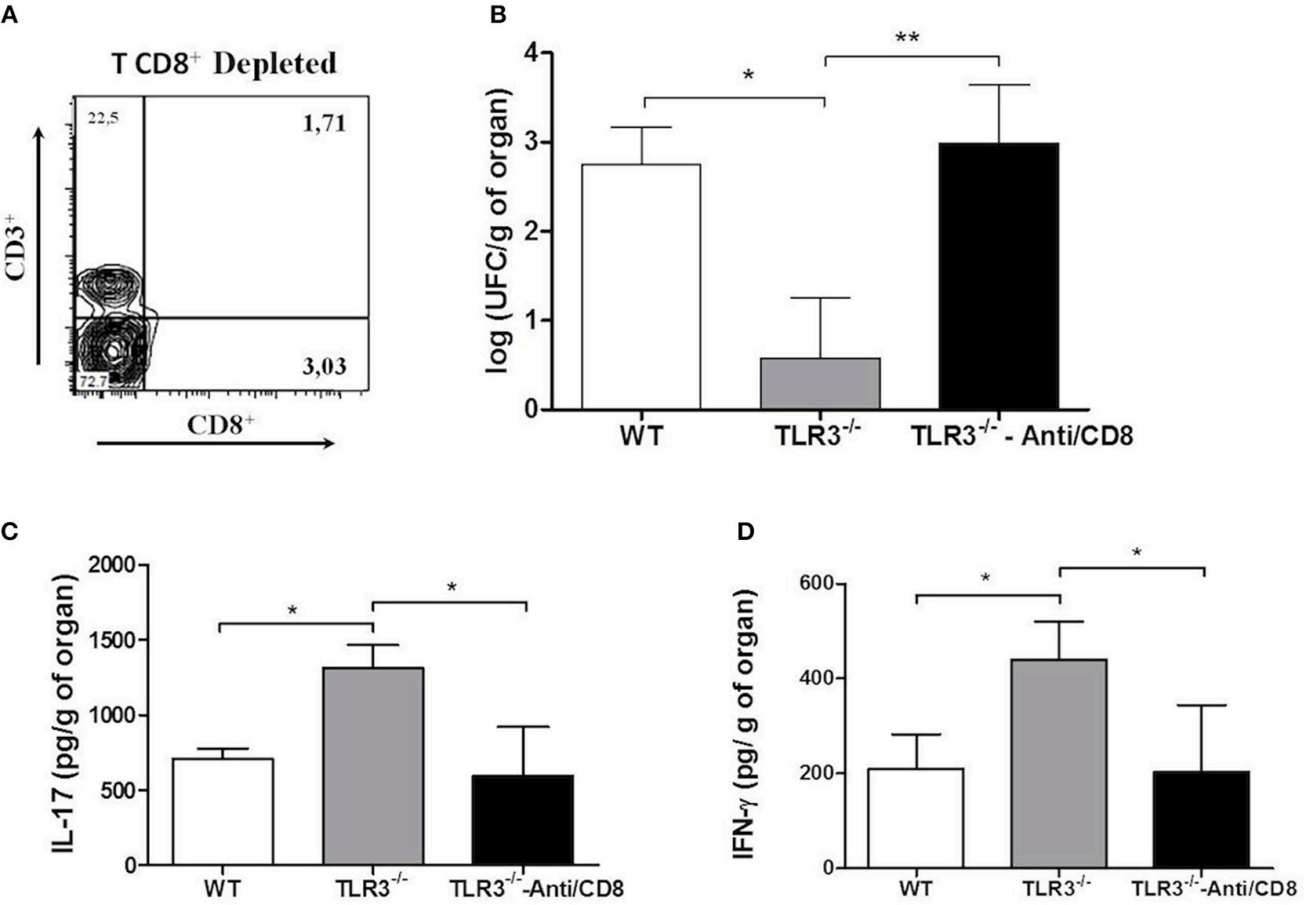
Absence of CD8^+^T cells induce an increase of CFU. WT and TLR3^−/−^ mice (7 animals/group) treated or not with anti-CD8 were infected with 1 × 10^6^ yeast cells of Pb18 strain. After 30 days, the lung were obtained and we evaluated: **(A)** depletion of CD8^+^T cells; **(B)** CFU; **(C)** IL-17 cytokine, and **(D)** IFN-γ. Dates are means of four independents experiments (*P <0.05 and **P <0.001).

As demonstrated above, our results suggest that CD8^+^T cells are important in the control of *Paracoccidioides brasiliensis* infection. So, to clear the participation of these cells in our model, the CD8^+^T cells were depleted from TLR3^−/−^ mice (Figure 6A) and the CFU and cytokines IL-17 and IFN-γ were measured. Our results showed a significant increase of fungal burden (Figure 6B) and a decreased of the cytokines IL-17 and IFN- γ (Figures 6C,D, respectively) in the absence of CD8^+^ T cells.

## Discussion

The activation of an adaptive immune response depends on the early innate events that occurred during some infections. These events are initiated by cells responsible for recognizing and responding to invading pathogens, such as macrophages (Hoebe et al., 2004). The binding of pathogens with PRRs is an efficient mechanism of control infectious diseases. However, in some cases these pathogens may subvert this efficient mechanism and induce an immune escape mechanism. Here, we observed that the bone morrow-derived macrophages from TLR3 KO mice killed the internalized yeast, showing fungicidal activity with NO production. During infection, NO production is one of defensive strategies used by immune cells. The absence of iNOS induced an increase of fungal burden in *Paracoccidioides brasiliensis* infection, demonstrating the importance of the NO in the control of infection (Bernadino et al., 2013; Parente et al., 2015). In accord with this, we demonstrated that macrophages play a crucial role in the regulation of fungal growth in the early stages of infection (Calich and Kashino, 1998; De Souza et al., 2015). In addition, another aspect that is essential in innate immunity against fungal pathogens is the presence of TLRs.

TLR2 and TLR4 have long been known to be involved in the recognition of fungi and have been implicated in the recognition of *Paracoccidioides brasiliensis* (Gersuk et al., 2006; Loures et al., 2009, 2010). More recently, studies have suggested that TLR3, an intracellular receptor, may also be involved in the control of *Aspergillus* infection (Shin and Lee, 2010; Carvalho et al., 2012). Carvalho and co-works demonstrated that TLR3^−/−^ mice infected with *A. fumigatus* conidia had increased pulmonary fungal burden and a higher incidence of dissemination. However, in opposition to this, in the present work, we observed for the first time that the absence of TLR3 induces the control of a virulent *Paracoccidioides brasiliensis* infection. We observed a decreased of fungal burden in the lungs of the TLR3^−/−^ mice, suggesting that the fungus can trigger TLR3 activation and thus modulates and escape of host immune response.

The phenotypic analysis of lymphocyte subsets in the lung was performed in knockout mice for TLR3 to analyze cytokine production. Our results showed an increased of CD8^+^ T cells in the lungs. In addition, our findings in the lung, demonstrated a higher amount of IL-17, IL-1β, IFN-γ, and IL-6 production. In accord with this, in experimental PCM, the role of CD4^+^ and CD8^+^ T cells in murine PCM was characterized, and the authors showed that, in pulmonar PCM, the fungal burden were primarily controlled by CD8^+^T cells (Chiarella et al., 2007; Loures et al., 2014). Here, we have also observed a cytotoxic mechanism when the infected macrophages were cultivated with CD8^+^T cells. In a previous study by our group (Jannuzzi et al., 2015), we showed the importance of CD8^+^T cells in experimental PCM after treatment with a scFv molecule specific to gp43, which is a specific glycoprotein from *Paracoccidioides brasiliensis*. Moreover, in experimental PCM is the first time that a study showed the cytotoxic effects of CD8^+^ T cells, mediated by granzimes and perforin production, that are responsible to destroying the infected cells (Wherry et al., 2013). In addition, previous studies showed the interaction between CD8^+^ T cells and IL-17 production (Gersuk et al., 2006). Th17 polarization in pulmonar PCM is associated with PMN-rich inflammatory reactions (Loures et al., 2009, 2010, 2011). Morais and coworkers suggested that CPG, a DNA that links with TLR9, induces the production of pro-inflammatory cytokines such as IL-1β and IL-6 (Morais et al., 2016). Additionally, it was recently shown that the cytokine IL-17 plays a key role in innate antifungal defenses, contributing to fungal clearance as observed in mucosal candidiasis (De Souza et al., 2015).

In agreement with these results, we observed a significant production of cytokines involved with pro-inflammatory activity, such as IL-17, IL-6 and IL-1β, in TLR3^−/−^ mice and we have also showed an increase of CD8^+^T cells that produce IFN- γ and IL-17. IFN-γ is a well-known cytokine in experimental PCM (Alegre-Maller et al., 2014). The significant increase in this cytokine that was observed in our model suggested a positive response after *Paracoccidioides brasiliensis* infection and that IFN-γ could participate in the clearing of fungus in the lungs of TLR3^−/−^ mice. However, we observed too, an increase of Treg cells in the lung of TLR3^−/−^ mice. The role of Treg cells is controversial. While some studies showed that these cells could depress the immune system, others, suggest that the presence of Treg cells, could be responsible to decrease the tissue damage, during an immune response (Mills, 2004; Pagliari et al., 2011). Fenolato et al. (2012), demonstrated that resistant mice against *Paracoccidioides brasiliensis* have more T reg cells, where they compared with the susceptible mice (Fenolato et al., 2012).

To clear the importance of the CD8^+^T cells and the cytokines in our model, we block these cells and after *Paracoccidioides brasiliensis* infection, we observed an increase of the fungal burden and a decrease of IL-17 and IFN- γ. These results, suggest that the fungus, in the absence of TLR3, induce an efficient cytotoxic effects of CD8^+^ T cells. In accordance, Alexandre and coworks showed that the cytotoxic immune response was a mechanism to control *Lacazia loboi* infection. They suggested that CD8^+^ T cells could play a role in the elimination of the fungus (Alexandre et al., 2017).

Although the mechanism by which TLR3 is modulating the response in experimental infection has been clarified, we can suggest that the fungus uses this receptor as an escape mechanism and that one of the pathways could be through the regulation of CD8 T cells. It has already been described in the literature the importance of these cells in the control of this infection and as TLR3 interferes in this response, as we saw in our results, it could be this way that the fungus uses to survive in the tissue. In addition, once intracellular TLR3 receptors are activated, other mechanisms are activated, such as the modulation of cytokine production, which could be helping in the modulation of CD8 T cells.

**Figure 7:**
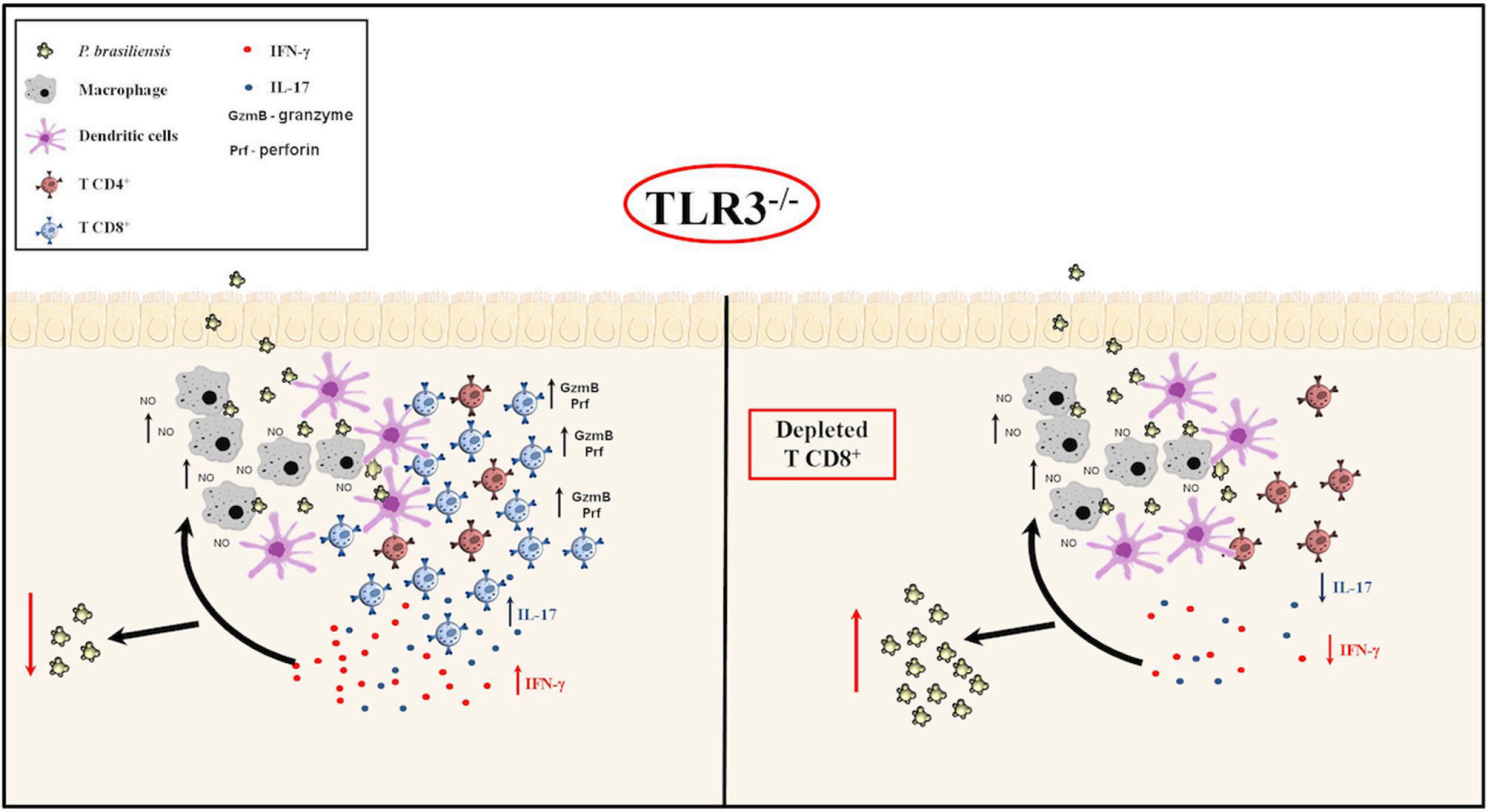
TLR3 in experimental PCM. Briefly, our results suggest that *in vitro*, BMDM produce greater NO in the presence of yeast, especially in the presence of IFN-γ. After activation of the adaptive immune response, *in vivo*, there was an increase in cytotoxic CD8 ^+^ T cells and IL-17 and IFN-γ production, with a decreased of *Paracoccidioides brasiliensis* yeast cells, as we can see in **(Left)**. On the other hand, when we depleted the CD8^+^ T cells in TLR3^−/−^ mice, we observed an increased of the CFU, but, a decreased of IL-17 and IFN-γ cytokines production **(Right)**.

In conclusion, we showed that the absence of TLR3, the NO production by macrophages, cytotoxicity mechanism, and an increase in CD8^+^T cells and pro-inflammatory cytokines could induce control of *Paracoccidioides brasiliensis* infections. This study highlighted the relevant role of TLR3, an essential receptor in innate immune responses, by effective inhibition of murine PCM. Our study results provided clarification on susceptibility to *Paracoccidioides brasiliensis* pulmonary infection, as we can see in the Figure 7.

## Author Contributions

The study was planned and wrote by KF. The development of the CFU, cytokine production and cytometry experiments was doneby GPJ. The phagocytosis test was done by GK and GPJ. The figures 1 and 2 were prepared by JdA. The nitric oxide production was done by LR. The cytotoxicity assay was co-designer and discussed with GP-AM, JdA, and CF. The authors SdA and KF discussed the results and all authors reviewed the manuscript.

### Conflict of Interest Statement

The authors declare that the research was conducted in the absence of any commercial or financial relationships that could be construed as a potential conflict of interest.
