# Supplementary material for: TLR3 Is a Negative Regulator of Immune Responses Against Paracoccidioides brasiliensis
Source: Front Cell Infect Microbiol. 2019 Jan 10;8:426. doi: 10.3389/fcimb.2018.00426 (PMC6335947; doi:10.3389/fcimb.2018.00426)
Supplement: Supplementary file 1 [file Image_1.pdf]

Material and Methods

Flow Cytometry Assay

To analyze the phenotype of the cells in the lung after infection (30 days), the total cells were obtained and analyzed by flow cytometry with a FACS Canto II (Becton Dickinson). To analyze DC and NK cells, the cells were stained with APC CD11c (N418), APC NK1.1 (PK136). To analyze the IL-17$^+$CD4$^+$ and IFN-γ+ CD4$^+$ T cells, the lungs cells were incubated with Brefeldin A for 12 hours. After that, the CD8$^+$T cells were stained with PerCP CD3e (145-2C11) and FITC CD4 (RM4-5) and then we used the fixation and permeabilization kit (eBioscience) according to the manufacturer's protocol. The FITC-IFN-γ and PE-IL-17 antibody were added and then the CD8$^+$T cells were analyzed by flow cytometer. All antibodies were obtained from BD Biosciences (San Jose, CA). The flow cytometry data were analyzed using FlowJo. Fluorescence-minus-one (FMO) tubes were used as additional controls.

Results

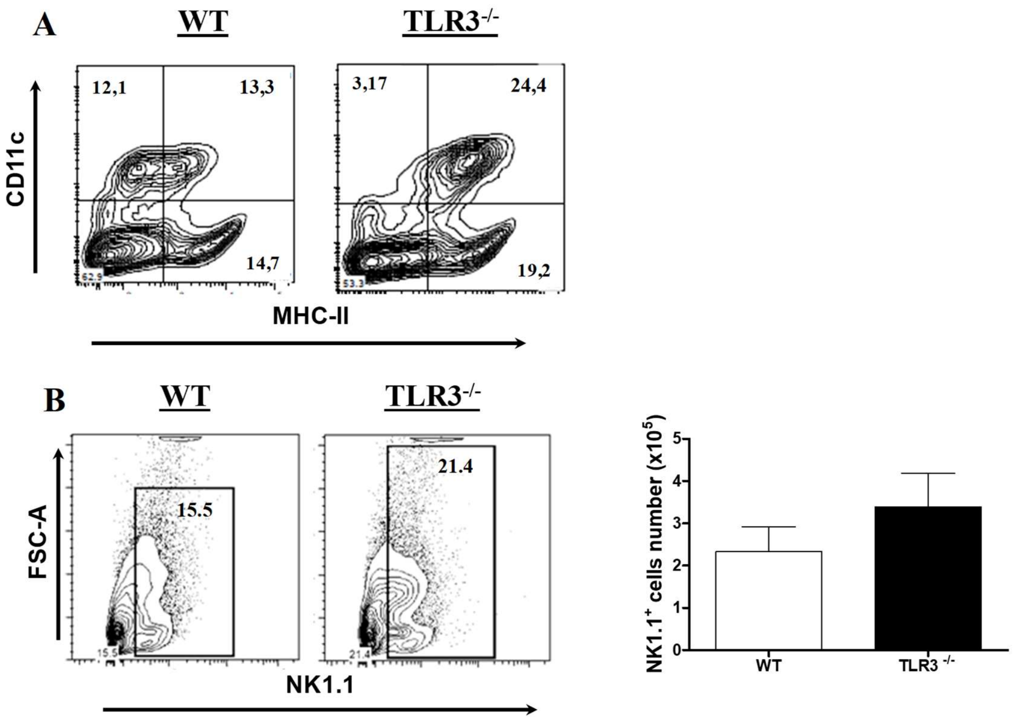

Supplementary Figure 1. DC and pulmonary NK cells do not show any difference between lineages. WT and TLR3$^{-/-}$ mice (seven/group) were infected with 1x10^6 of Pb18 yeast for 30 days and (A) total CD11c$^+$/MHC-II$^+$ and (B) NK cells from lung tissue were investigated by flow cytometry.

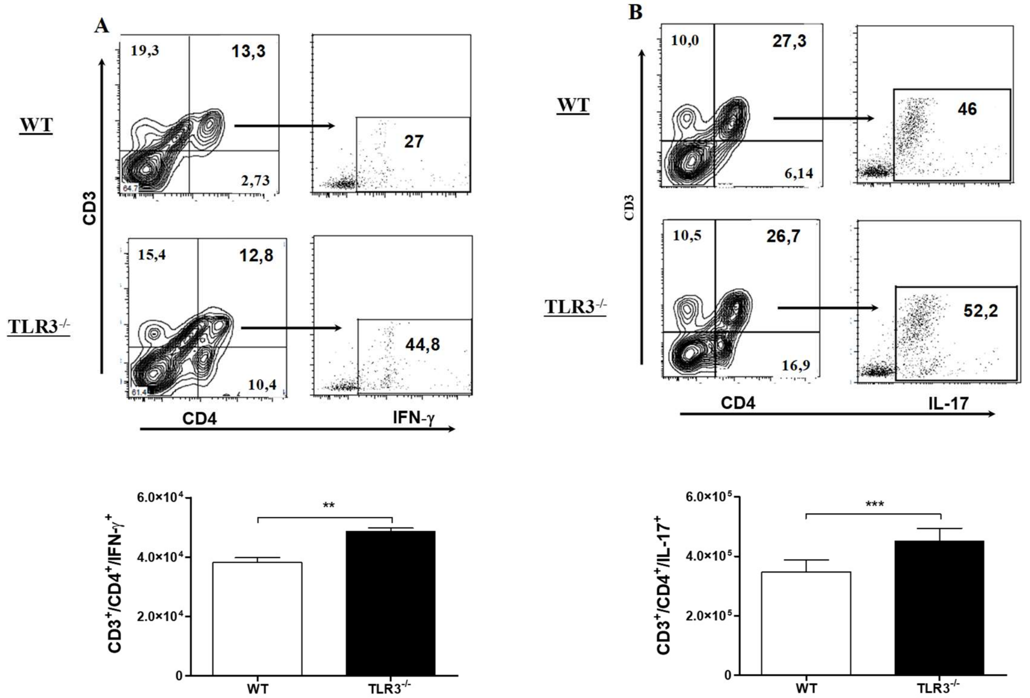

Supplementary Figure 2. CD4$^+$ T cells increased in the lung of TLR3$^{-/-}$ mice. WT and TLR3$^{-/-}$ mice (seven/group) were infected with 1x10^6 of Pb18 yeast for 30 days and total pulmonary CD4+T cells (A) IFN-g and (B) IL-17 intracellular cytokines produced were investigated by flow cytometer and the results were measured from mix of lung from seven animals. (* P< 0.05, **P <0,001 and ***P<0.0001).
